# Bacterial communities in the gastrointestinal tract segments of helminth-resistant and helminth-susceptible sheep

**DOI:** 10.1186/s42523-022-00172-1

**Published:** 2022-03-14

**Authors:** Erwin A. Paz, Eng Guan Chua, Shamshad Ul Hassan, Johan C. Greeff, Dieter G. Palmer, Shimin Liu, Binit Lamichhane, Néstor Sepúlveda, Junhua Liu, Chin Yen Tay, Graeme B. Martin

**Affiliations:** 1 UWA Institute of Agriculture, The University of Western Australia, Perth, 6009 Australia; 2 Helicobacter Research Laboratory, The Marshall Centre for Infectious Disease Research and Training, School of Biomedical Sciences, The University of Western Australia, Perth, 6009 Australia; 3 Department of Primary Industries and Regional Development, South Perth, 6151 Australia; 4 Departamento de Produccion Agropecuaria, CTI-Carne-CEBIOR-BIOREN, Universidad de La Frontera, Av. Francisco Salazar 01145, Temuco, Chile; 5 College of Animal Science and Technology, Nanjing Agricultural University, Nanjing, 210095 China; 6 CY O’Connor ERADE Village Foundation, P.O. Box 1, North Dandalup, WA 6207 Australia

**Keywords:** Sheep, Microbiome, Helminth, Faecal-egg count, 16S rRNA gene sequencing, Nematode, Short-chain fatty acids

## Abstract

**Background:**

Helminth parasitism is a world-wide problem in livestock industries, with major impacts on health, welfare and productivity. The role of the gut microbiota in host-helminth interactions in ruminants has been extensively examined and the present study added to this body of knowledge by assessing the effects of resistance and susceptibility to helminth infection in the gastro-intestinal tract (GIT). Australian Sheep Breeding Values (ASBVs) for faecal egg count (FEC) were used to select the 10 highly helminth-susceptible (High-FEC) and 10 highly helminth-resistant (Low-FEC) sheep. FEC status was confirmed during the experiment. Using samples from the faeces and the lumen of the rumen, abomasum, duodenum, jejunum, ileum, caecum, and colon, DNA was extracted and used for 16 rRNA gene amplicon sequencing.

**Results:**

The most frequent genera identified along the GIT were *Eubacterium*, *Oscillibacter,* and *Ruminococcus*. Intersectoral-specialization zones were identified along the GIT, with the duodenum displaying major differences between the High-FEC and Low-FEC animals in values for alpha and beta diversity. After taking all samples into account and adjusting for GIT segment, the High-FEC and Low-FEC sheep differed significantly for four genera *Butyrivibrio*, *Mycoplasma*, *Lachnoclostridium* and *Succiniclasticum*. In the duodenum, the abundances of *Aminipila*, *Lachnoclostridium* and *Mogibacterium* differed significantly between the High-FEC and Low-FEC sheep. In the ileum, on the other hand, the genus *Mycoplasma* was significantly depleted in the Low-FEC group.

**Conclusions:**

The gastro-intestinal microbial profile varies widely between helminth-resistant and helminth-susceptible sheep. Each GIT section appears to support a particular bacterial composition leading to inter-sectoral differences among the various microbial communities. The microbial populations were most rich and diverse in the duodenum of helminth-resistant sheep, comprising bacterial genera that generally ferment carbohydrates. This observation suggests that helminth-resistant sheep can reorganize the duodenal microbiome taxa which may restrict the development of parasites.

**Supplementary Information:**

The online version contains supplementary material available at 10.1186/s42523-022-00172-1.

## Background

Gastrointestinal helminth infection has a major economic impact in sheep industries worldwide—for example, for Australia in 2015, Meat and Livestock Australia estimated an annual loss of AUD436 million [1]. In the sheep of Western Australia, the major problem is infection during winter and early spring, with *Teladorsagia circumcincta*, found mainly in the abomasum, and *Trichostrongylus colubriformis* in the small intestine, particularly in the duodenum [2, 3]. Clinical signs associated with high levels of *T. circumcincta* and *T. colubriformis* infection include loss of appetite, rapid weight loss, profuse watery diarrhoea and hypoproteinaemia [4]. Other helminth species are also found, such as *Chabertia ovina*, *Oesophagostomum venulosum* and *Haemonchus contortus*, but they are less prevalent in this environment [5].

To mitigate helminth infection in their flocks, farmers worldwide have often resorted to frequent and intensive use of anthelmintic drugs, with the unfortunate outcome being the development of helminth populations that are resistant to one or more classes of parasiticide [6–8]. These problems have elicited global efforts in genetic selection using faecal egg count (FEC) as the phenotypic trait [9–11], to produce sheep that are naturally resistant to helminth infection. One example is the ‘Rylington Merino’ flock that was established in 1998 and is currently the most worm-resistant Merino flock in Australia [12]. In addition, the level of resistance to helminths varies within and between sheep genotypes, reflecting genetic variation in the production of immunoglobulin A (IgA) that is specific to helminth antigens, leading to variation in helminth survivability [13, 14].

There has been an increased interest in how microbial communities in the gastro-intestinal tract (GIT) are affected by parasite infection, raising the possibility of new avenues for investigating the drivers of gut homeostasis. For example, it was recently reported that the severity of infection with *H. contortus* in sheep is related to the faecal microbiota, implying a supporting role for gut microbes in modulating host resistance to infection [15]. Moreover, such observations suggest that, in the host, the intricate relationship between the immune system and the gut microbiome might be involved in the response to helminth infection [16]. In summary, it might be possible to identify particular bacterial communities that can be used to mitigate or control parasitic infection [17]. We therefore tested whether the structure and composition of the microbial communities along the GIT are affected by variation in genetic resistance to helminths, and identified specific microbial communities that are associated with helminth resistance. We used sheep with low (*n* = 10; Low-FEC) and high (*n* = 10; High-FEC) breeding values (genetic potential) for FEC and studied the GIT microbial populations using 16S rRNA gene amplicon sequencing.

## Results

### Parasitological differences between High-FEC and Low-FEC groups

The data for breeding value (ASBV), FEC and worm burden for the two genotypes are shown in Additional file 1: Table S1. The average values for cumulative FEC were 1940 ± 1120 eggs/g in the High-FEC group and 410 ± 423 eggs/g in the Low-FEC group (*p* = 0.018). This fourfold difference was a reflected in the average values for ASBV (High-FEC 35 ± 14 versus Low-FEC − 66 ± 4) and average values for worm burden (High-FEC 36,844 ± 23,733 worms versus Low-FEC 10,875 ± 12,320 worms). These observations validate the considerable divergence to helminth infection between the experimental groups.

### General analysis of microbial communities along the GIT

As shown in Additional file 2: Table S2, a total of 13,100,180 raw reads were sequenced from the 16S V3-V4 amplicons generated from the faecal material and the luminal samples from the seven GIT segments of the 20 sheep. By trimming, merging of overlapping paired-end reads, and filtering of sequences < 400 bp, the number of reads was reduced to 3,013,423 sequences, ranging from 4,751 to 62,456 sequences per sample, and with an average sequence length of 449 ± 4 bp. These sequences were converted into 328 OTUs at 97% sequence identity, revealing 14 phyla, 22 classes, 27 orders, 39 families and 59 genera after taxonomic classification.

**Fig. 1:**
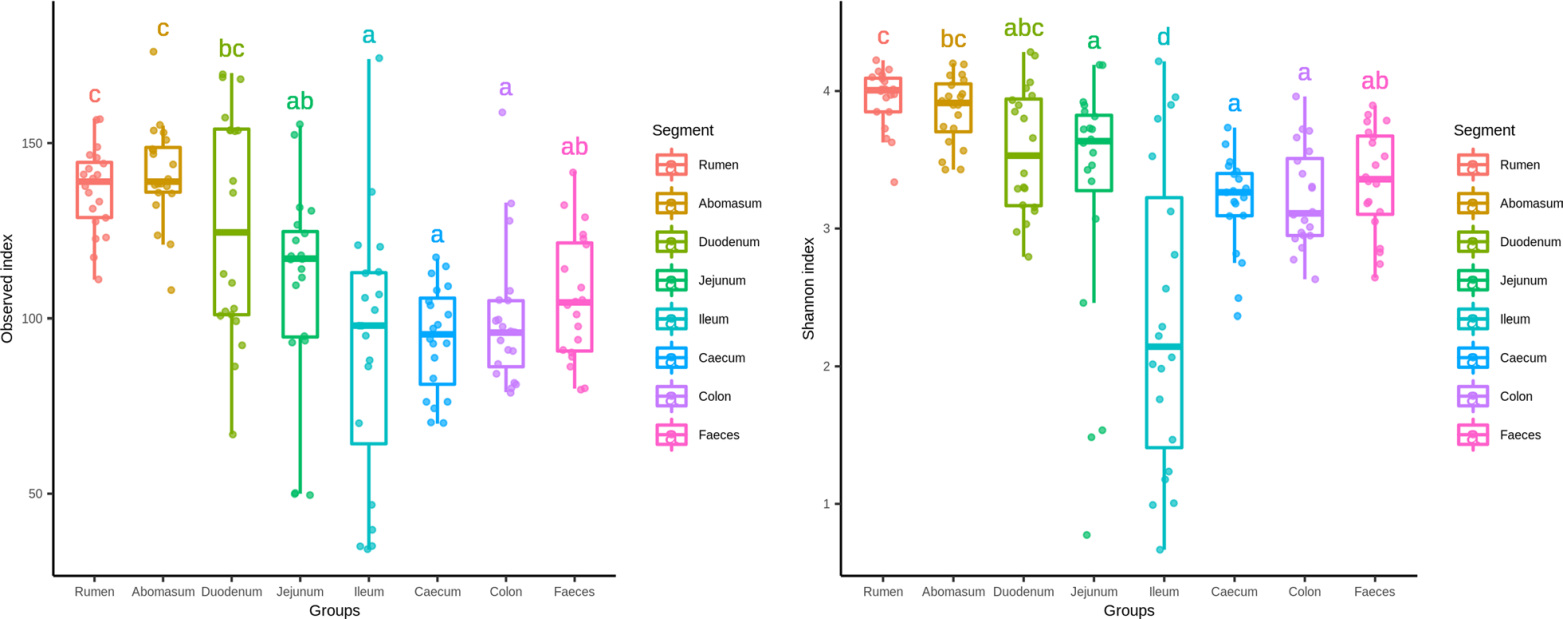
Differences in alpha diversity values, tested by ANOVA, among gastro-intestinal tract (GIT) segments from High- and Low-FEC sheep. ^a,b,c^Means followed by different letters above the graphs indicate statistically significant differences (*p* < 0.05). Graphs that share the same letter do not differ significantly

**Fig. 2:**
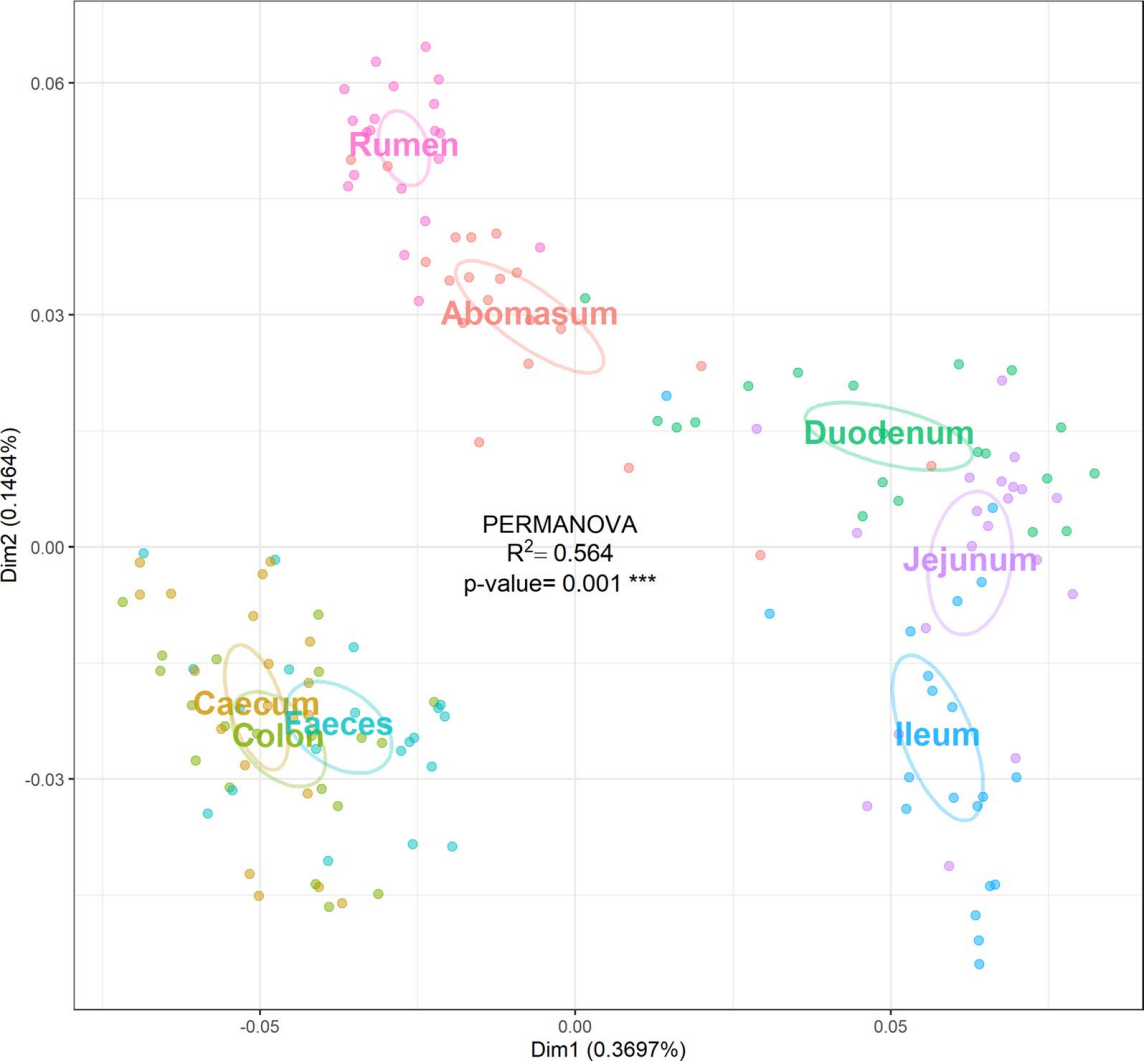
Principal coordinates analysis (PCoA) based on weighted UniFrac distance matrix of samples collected along the gastro-intestinal tract (GIT) of High- and Low-FEC sheep

**Fig. 3:**
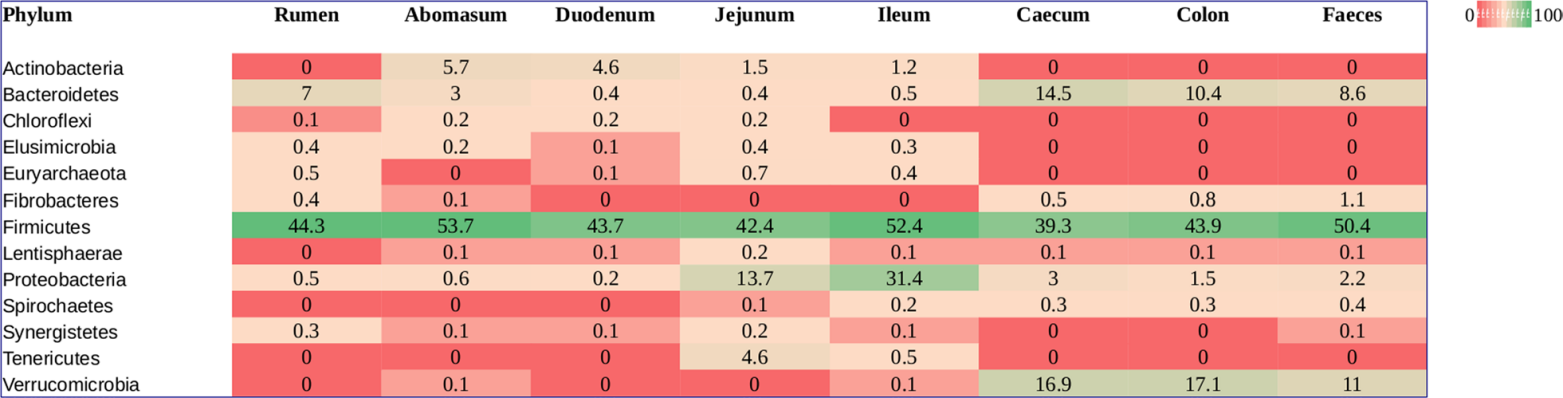
Heatmap showing significant differences in bacterial abundance among gastro-intestinal tract (GIT) segments at phylum level, based on ANCOM analysis. Each value represents the median relative abundance in percentage

**Fig. 4:**
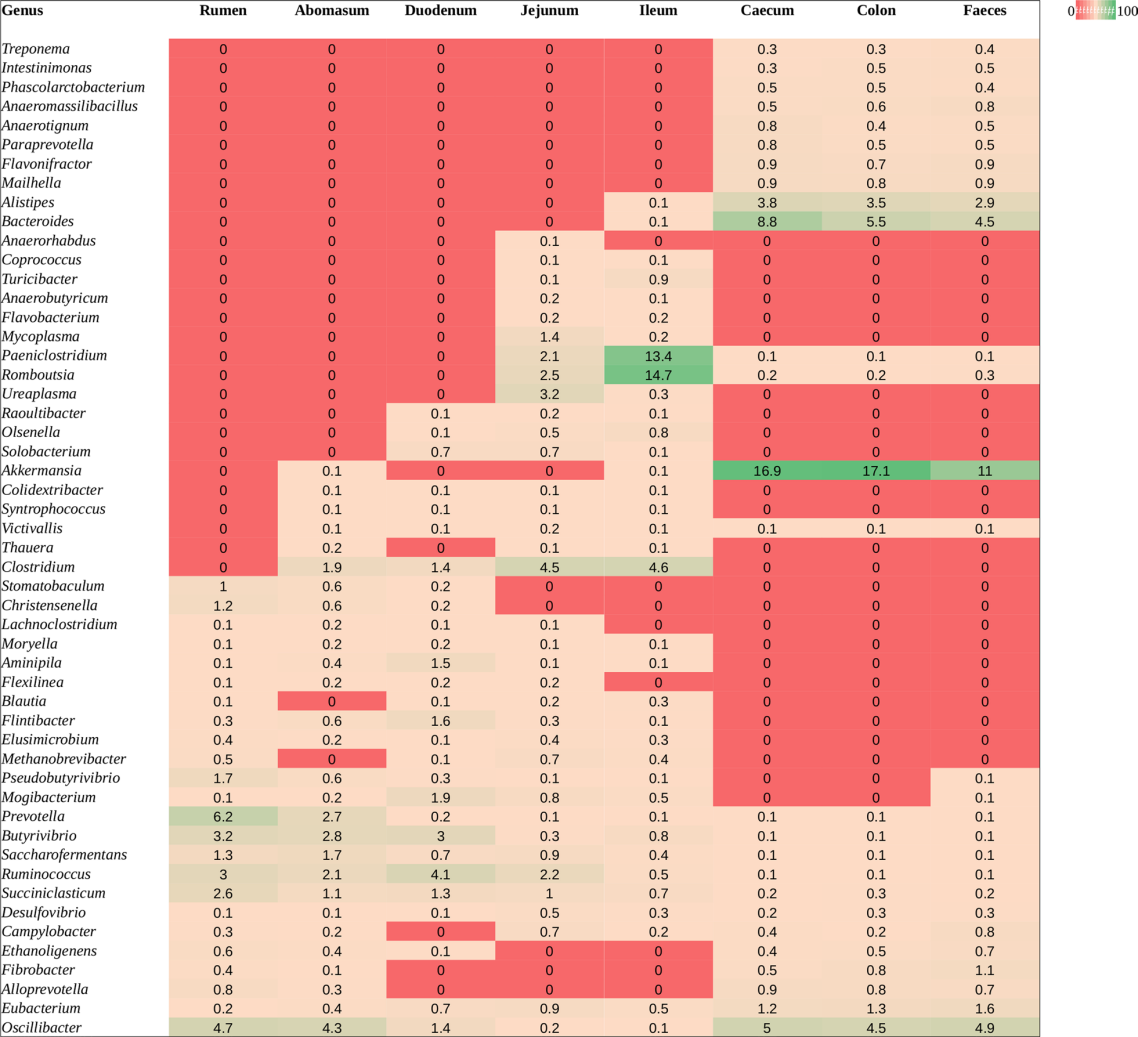
Heatmap showing significant difference in bacterial abundance among gastro-intestinal tract (GIT) segments at genus level, based on ANCOM analysis. Each value represents the median relative abundance in percentage

OTU richness and alpha diversity (Shannon index) of the microbial communities present in each GIT segment were calculated for High- and Low-FEC samples and statistical differences among the GIT segments of all sheep were assessed using ANOVA and Tukey’s post-hoc multiple comparisons analysis (Fig. 1 and Additional file 3: Table S3). The abomasum, rumen and duodenum contained the richest and most diverse populations, whereas the ileum produced the lowest values for both OTU richness and Shannon index (Fig. 1). Figure 2 shows significant differences in taxonomic composition between GIT segments (PERMANOVA, R^2^ = 0.564, *p* = 0.001). Principal coordinates analysis (PCoA) based on the weighted UniFrac distance presented a plot with a tight cluster containing the colon, caecum and faeces, with the rumen, abomasum, duodenum, jejunum and ileum in a different group with more scatter (Fig. 2). The segregation of the microbial communities was also evident in the contrast between the gastric (i.e., rumen and abomasum) and small intestine (i.e., duodenum, jejunum and ileum) compartments. Additional pairwise comparison revealed significant differences among all the GIT segments, except between caecum and colon (Additional file 6: Table S6). Firmicutes was the most abundant and ubiquitous phyla throughout the tract, followed by Bacteroidetes and Proteobacteria (Fig. 3). At genus level, *Prevotella*, *Butyrivibrio*, *Saccharofermentans*, *Ruminococcus*, *Succiniclasticum*, *Desulfovibrio*, *Eubacterium*, and *Oscillibacter* were the most common and ubiquitous (Fig. 4). The comparison of GIT segments shows that some phyla and genera are present or absent at specific sites and the most prominent differences are between the first part of the GIT (rumen to ileum) and the large intestine (caecum and colon). For example, Actinobacteria, Chloroflexi, Elusimicrobia, Euryarchaeota, Synergistetes, and Tenericutes are present exclusively in the first part of the GIT (Fig. 3). It should also be noted that eight genera (i.e. *Treponema*, *Intestinimonas*, *Phascolarctobacterium*, *Anaeromassilibacillus*, *Anaerotignum*, *Paraprevotella*, *Flavonifractor*, and *Mailhella*) were absent from the first part of the GIT, and 26 genera were absent from the large intestine (Fig. 4). The ANCOM test revealed 13 phyla and 52 genera that differed significantly among the GIT segments (Additional file 5: Table S5). Further statistical analysis, with each significant taxon compared among pairs of GIT segments is presented in Additional file 7: Table S7.

### Analysis of the bacteria communities present in the High-FEC and Low-FEC groups

**Fig. 5:**
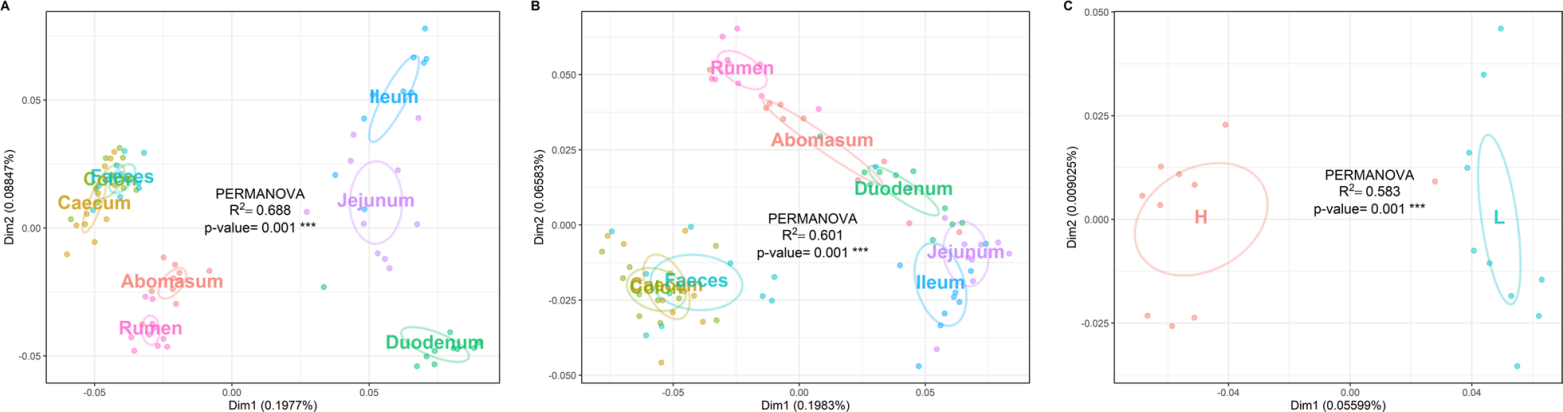
Principal coordinates analysis (PCoA) based on weighted UniFrac distance matrix data collected along the gastro-intestinal tract (GIT) of **A** the High-FEC group, **B** the Low-FEC group, **C** the High-FEC and Low-FEC groups in the duodenal segment

OTU richness and alpha diversity (Shannon index) were calculated for microbial communities present in each GIT segment, within and between FEC groups (Table 1). Interestingly, when the High-FEC and Low-FEC groups were compared, the only significant difference (*p* < 0.001) was the greater alpha diversity values for OTU richness and Shannon index in the duodenum of Low-FEC sheep compared to High-FEC. To avoid the potential confounding effect of FEC status, beta diversity across GIT segments was investigated separately for samples collected from High- and Low- FEC sheep. For the High-FEC group, our PCoA based on the weighted UniFrac distance metric (Fig. 5A) presented a plot with two tight clusters, one containing the rumen and abomasum samples, and the other containing the caecum, colon and faecal samples (PERMANOVA, R^2^ = 0.688, *p* = 0.001). Another group, comprising duodenum, ileum and jejunum, were more dispersed and well differentiated. For the Low-FEC group (Fig. 5B), there was a similar clustering effect (PERMANOVA, R^2^ = 0.601, *p* = 0.001), except that the duodenal samples had shifted towards the rumen-abomasum cluster, suggesting a different structure of duodenal microbiota in this group of animals. This observation prompted another round of PCoA to estimate differences in microbiota composition between the High-FEC and Low-FEC groups in the duodenum (Fig. 5C). The clustering differed significantly between the FEC groups (R^2^ = 0.583, *p* = 0.001). No differences were found for any of the other sites.

**Table 1:** Comparison of alpha diversity between High-FEC and Low-FEC groups in each gastro-intestinal tract segments

Segment/Group	OTU richness (mean ± SD)		*p* values	Shannon index (mean ± SD)		*p* values
	High-FEC	Low-FEC		High-FEC	Low-FEC	
Rumen	139 ± 14	133 ± 9	0.265	5.7 ± 0.4	5.7 ± 0.3	0.591
Abomasum	141 ± 15	142 ± 10	0.867	5.5 ± 0.3	5.6 ± 0.4	0.475
Duodenum	103 ± 24	149 ± 22	< 0.001	4.6 ± 0.4	5.6 ± 0.5	< 0.001
Jejunum	103 ± 33	113 ± 27	0.456	4.4 ± 1.6	5.0 ± 1.1	0.375
Ileum	80 ± 31	104 ± 41	0.151	2.7 ± 1.3	3.9 ± 1.7	0.091
Caecum	92 ± 15	98 ± 16	0.366	4.6 ± 0.6	4.6 ± 0.4	0.735
Colon	103 ± 26	99 ± 15	0.679	4.7 ± 0.6	4.6 ± 0.5	0.844
Faeces	109 ± 23	106 ± 16	0.796	4.9 ± 0.6	4.8 ± 0.5	0.694

The ANOVA with Tukey's post-hoc test was used to assess the differences among groups

FEC, faecal egg count; SD, standard deviation

Table 2 presents the bacterial genera that differed significantly between the High-FEC and Low-FEC groups, for the total GIT and the individual GIT segments, as determined by ANCOM. In the duodenum, two genera, *Aminipila* and *Lachnoclostridium*, were significantly depleted, whereas *Mogibacterium* was enriched, in the High-FEC samples compared to the Low-FEC samples. In the ileum, on the other hand, there were significantly fewer mycoplasma in the Low-FEC group than in the High-FEC group. When taking all the samples into account, after adjustment for GIT segment, four bacterial genera differed significantly between the High-FEC and Low-FEC groups: the average relative abundances of *Succiniclasticum* and *Butyrivibrio* were significantly lower*,* whereas average relative abundances for *Lachnoclostridium* and *Mycoplasma* were significantly greater, in the High-FEC group compared with the Low-FEC group.

**Table 2:** Bacterial genera showing significant differences in abundance between individual gastro-intestinal segments, as well as the whole gastro-intestinal tract (GIT), of High- and Low-FEC sheep

Segment	High-FEC (mean ± SD)	Low-FEC (mean ± SD)
Duodenum		
*Aminipila*	2.8 ± 8.2	49.8 ± 75.2
*Lachnoclostridium*	0.2 ± 0.6	4.7 ± 2.6
*Mogibacterium*	51.5 ± 40.8	14.8 ± 10.2
Ileum		
*Mycoplasma*	6.1 ± 8.9	0.7 ± 2.2
Whole GIT		
*Succiniclasticum*	10.7 ± 20.3	21.6 ± 36.4
*Lachnoclostridium*	0.9 ± 1.8	2.1 ± 4.4
*Butyrivibrio*	19 ± 41	38.7 ± 89.8
*Mycoplasma*	6.5 ± 36.7	0.3 ± 1

SD: standard deviation

## Discussion

This study compared GIT bacterial communities in helminth-resistant and helminth-susceptible sheep, in which there was a 400% difference in FEC and a 300% difference in parasite burden, following natural grazing under natural Mediterranean conditions [5]. The dominant helminths are *Teladorsagia* spp. in the abomasum and *Trichostrongylus* spp. in the duodenum. There were no significant differences between the groups of sheep for alpha diversity measurements in the rumen, abomasum, jejunum, ileum, caecum, colon or faeces. However, in the duodenum, OTU richness and Shannon diversity were lower in the susceptible sheep than in the resistant sheep. In general ecological terms, greater diversity in a community indicates a more stable and favorable environment. One possible explanation is that the duodenum of helminth-resistant sheep supports a better-adapted microbiome in which it may be difficult for helminths to thrive. Other factors that affect microbiome diversity within the gut include genetic background, physiological condition, diet, and health status [18]. In helminth-susceptible sheep, damage to the GIT by the parasite may inflame the duodenal mucosa, increasing protein leakage and changing the pH at this site, perhaps favoring the excessive growth and development of some bacterial species at the expense of others [19, 20]. The differences in alpha diversity observed across GIT segments were significant when both High- and Low-FEC groups were considered. Observed OTUs and Shannon index in the duodenum and jejunum (i.e., small intestine) were higher than in the large intestine. In this context, the lowest richness/diversity values were found in the ileum, suggesting that proliferation of the micro-flora is restricted in this segment of the small intestine, because of the high concentrations of bile, salts and digestive enzymes [21].

Three genera in the duodenum differed significantly in their abundance between the resistant and susceptible sheep—the *Aminipila*, *Lachnoclostridium* and *Mogibacterium*—the first two of which have only been recently identified as new taxa and were found abundant in the helminth-resistant group. In brief, *Aminipila,* isolated from cattle waste in 2018, has been associated with the degradation of L-arginine, L-lysine and L-serine, and with the production of short-chain fatty acids (SCFAs), particularly acetate and butyrate [22]. *Lachnoclostridium*, is a new genus that includes a number of new species that have been identified in the human gut in relation to colorectal tumorigenesis [23]. This genus was also significant in the helminth-resistant sheep when both High- and Low-FEC groups were examined along the whole GIT. They are associated with the production of butyrate [24, 25] but there is currently no information about their role or function in the sheep GIT. Recent evidence has suggested that specific fermenting bacteria are linked to intestinal homeostasis by the production of SCFAs metabolites associated to host metabolism, intestinal functions, and immunity system. For example, lower levels of butyrate were found in faeces of equines infected with high parasite burdens compared to the low parasite burden group [26]. Moreover, greater numbers of *Mogibacterium* were found in the duodenum of the helminth-susceptible group than in the helminth-resistant group, perhaps in association with disrupted gut conditions. This genus is abundant in humans with colorectal cancer, as well as in piglets fed a control formula that did not include beneficial prebiotics [27, 28]. Interestingly, the analysis of individual segments showed in the ileum of helminth-susceptible sheep a significant abundance of mycoplasma as well as along the whole GIT. Mycoplasmas are the smallest prokaryotic group found in nature and, as a result, they generally need host cells to supply biochemical compounds [29]. Many members of this genus have been identified in small ruminants, but only a few are considered to be clinically relevant [30]. At this point, the role of mycoplasmas in GIT responses to helminth infection is difficult to assess. Greater numbers of *Succiniclasticum* and *Butyrivibrio* differed in relative abundance in the helminth-resistant sheep along the whole GIT. This group mainly ferments carbohydrates to produce propionate, butyrate and formate. The genus *Succiniclasticum*, previously identified in the cow rumen, can only ferment succinate to produce propionate [31]. The members of the *Butyrivibrio* genus can produce, characteristically, butyrate and formate from a variety of carbohydrates [32]. In a previous study, Li and coauthors observed that helminth infection decreases the relative abundance of *Butyrivibrio* in goats infected with *H. contortus* [19]. These observations align with our finding of an increased abundance of butyrate-producing bacteria in helminth-resistant sheep, suggesting that SCFA-producing bacteria might be involved in the regulation of the helminth infection. Particularly butyrate, could regulate the helminth infection by reducing the expression of potent inflammatory molecules including tumor necrosis factor (TNF) and inducible nitric oxide synthase (iNOS) and regulate the recruitment and migration of immune cells (leukocyte, macrophages, dendritic cells, and T cells) to the infection site [33].

Microbial composition varied substantially among rumen-abomasum, small intestine, and large intestine, particularly with three genera: (i) *Eubacterium*, a genus that includes a wide spectrum of species [34]; (ii) *Oscillibacter*, an abundant genus in the faeces of free-grazing sheep (although with low values in the jejunum and ileum) that is linked to the production of butyric acid and alpha-linolenic acid [35]; and (iii) *Ruminococcus*, a genus of cellulose degraders found in various species [36]. We consider these three genera to be members of the core bacterial communities throughout the GIT in High- and Low-FEC sheep. These genera belong to the Firmicutes phylum and most of them can decompose fibre and cellulose [37]. Other studies in Small-Tailed Han and Chinese Mongolian sheep also describe *Prevotella* as one of the most important and ubiquitous genera throughout the sheep GIT [21, 38].

## Conclusions

We evaluated and identified the microbiome from faeces and seven different sections along the sheep GIT and compared the populations in helminth-resistant and helminth-susceptible sheep. Every section of the sheep GIT supports quite specific bacterial communities and the distribution of communities is affected by resistance to parasite infection. This effect is particularly evident at the major sites of infection—specifically, the duodenum and the ileum. We conclude that helminth-resistant sheep support a more diverse variety of microbial communities and promote species that favor the production of SCFAs that could be involved in the processes that confer resistance to infection.

## Methods

### Animals and experimental design

This experiment was undertaken at the Katanning Research Station of the Department of Primary Industries and Regional Development (DPIRD) in Western Australia. It is located in a winter rainfall region with warm dry summers and cold wet winters. The most common and important worm species in this environment, *T. circumcincta* and *T. colubriformis,* can cause problems during winter, spring and up to early summer.

The sheep in this experiment were a sub-sample from a larger experiment [5] involving 986 lambs that had been born in July–August 2016 and weaned in November 2016. At weaning, the lambs were faecal sampled and administered a broad-spectrum oral anthelminthic (Monepantel; 1 mL/10 kg body weight). FEC was determined using the modified McMaster technique [39] with a sensitivity of 40 eggs/g faeces. Male and female lambs were separated at weaning and placed in two similar paddocks at a stocking rate of about 10 sheep/hectare. The pasture composition of the two fields during winter and spring was similar—primarily various *Trifolium* spp. and annual grass species with cape weed (*Arctotheca calendula*). In addition to the pasture, the sheep were supplemented with oaten hay ad libitum plus a supplement (500 g per sheep daily) of mixed barley and lupin grain, weaning until the end of the experiment in September 2017.

The FEC data at weaning, along with the completed pedigrees and the FEC data from previous generations, were submitted to Sheep Genetics [40], the Australian National Genetic Evaluation Scheme for sheep, to obtain the Australian Sheep Breeding Values (ASBVs) for FEC. ASBVs are estimated using Best Linear Unbiased Prediction mixed model methodology [41]. It uses all available pedigree information and adjusts the data for any factors that can affect the phenotypic measurement, such as management groups, animal sex, animal age, and birth status. The ASBV thus provides an unbiased prediction of the genetic worth of an animal, so it is the most accurate way to genetically differentiate between individuals and to identify genetically superior sheep. Additional information regards ASBVs can be found at following website operated by Meat and Livestock Australia [40].

Before the start of the experiment, the ASBVs for FEC were used to identify the most helminth-resistant and most helminth-susceptible animals: 100 males and 100 females (50 resistant and 50 susceptible sheep for each sex). These 200 sheep were maintained with their contemporaries for the duration of the experiment. From February 2017, they were faecal sampled monthly (Additional file 1: Table S1) to measure the increase in FEC up to slaughter in September 2017. In September 2017, we identified 18 sheep that had shown consistently high FEC and 20 sheep that had shown consistently low FEC, from February. These 38 sheep were transported to the laboratory in Albany in Western Australia. At 24 h prior to sacrificed, they were all confirmed to be in good health and were placed in individual sanitized pens with free access to water. After slaughter, the gut was immediately removed and luminal samples were collected from the rumen, abomasum, duodenum, jejunum, ileum, caecum, and colon. Faecal material was also sampled from each animal. All samples were stored at − 80 °C. From the 38 sheep, the 10 with the lowest FEC and 10 with the highest FEC were identified, and their luminal contents were analysed.

### DNA extraction

DNA was extracted using the QIAamp® Fast DNA Stool Mini kit (Qiagen, Germany) with substantial modifications. In brief, 250 mg of each luminal or faecal sample was mixed individually with 1 mL of InhibitEX buffer and incubated at 95 °C for 5 min. The resultant supernatant was transferred to a new tube included 600 µL buffer AL and 25 µL of proteinase K incubated for 1 h at 70 °C. The cell lysate was thoroughly mixed with one volume of phenol:chloroform:isoamyl alcohol solution (25:24:1) for 1 min and centrifuged at 10,000 × *g* for 5 min. After recovering the aqueous phase, the process was repeated. Subsequently, the aqueous phase was transferred into a new 1.5 mL tube before adding an equal volume of chloroform:isoamyl alcohol solution (24:1). The mixture was vortexed for 1 min and centrifuged at 10,000×*g* for 5 min. The aqueous phase was again transferred into a new 1.5 mL tube, after which was added with two volumes of ice-cold 95% (v/v) ethanol to precipitate the DNA. The resulting DNA pellet was washed with 70% (v/v) ethanol and resuspended in 50 µL Tris–EDTA buffer (10 mM Tris–HCL, pH 8.0, 1 mM EDTA). DNA integrity was checked on a 1% (w/v) agarose gel electrophoresis and the amount of DNA was quantitated using a Nanodrop spectrophotometer.

### 16S rRNA gene library preparation

The V3-V4 hypervariable region of the 16S rRNA gene was amplified using the primer sets as specified in Illumina’s 16S metagenomic sequencing library preparation protocol [42]. In the initial round of PCR amplification, the reaction mixture contained 30 ng of input DNA, 2 units of Taq DNA polymerase (New England Biolabs, United States), 10 mM dNTP, 10 µM each of the forward and reverse primers, and 1 × standard Taq reaction buffer. The PCR conditions comprised an initial denaturation at 95 °C for 30 s, followed by 29 amplification cycles comprising denaturation (95 °C for 30 s), annealing (55 °C for 40 s), extension (68 °C for 1 min), and a final extension at 68 °C for 5 min. PCR amplicons were visualized using gel electrophoresis on a 1.5% (w/v) agarose gel. Following purification of PCR products using AMPure XP beads (Beckman Coulter, United States), indexing PCR was performed using Nextera® XT Index kit (Illumina, United States) according to manufacturer’s instructions. The libraries were sequenced on an Illumina MiSeq instrument using the 2 × 300 bp paired-end v3 chemistry.

### Data analysis

Raw sequencing data were subjected to quality and adapter trimming using the bbduk.sh command available in BBTools (https://jgi.doe.gov/data-and-tools/bbtools/) with the following parameters: qtrim = r; trimq = 20; ktrim = r; k = 23; mink = 11; tpe; tbo; hdist = 1; and minlen = 200. After merging of overlapping paired-end reads using MeFiT software with default parameters, sequences with less than 400 bp were filtered [43]. The remaining sequences were subjected to de novo* unoise* clustering at 97% sequence identity threshold by running the *micca otu* command in Micca software (version 1.7.2) to obtain operational taxonomic unit (OTU) sequences [44]. Taxonomic classification of each representative OTU sequence was performed using the Bayesian LCA-based taxonomic classification method against the NCBI RefSeq 16S rRNA database [45], where the acceptance of a taxonomic assignment at each level was based on a minimum confidence score of 80. The OTU table and the taxonomic information are available in Additional file 4: Table S4.

Alpha and beta diversities were estimated using microbiomeSeq R package (https://github.com/umerijaz/microbiomeSeq) and QIIME v1.9.1 [46]. Following rarefaction at the sequence depth level of 1722, alpha diversity was analyzed on the basis of OTU richness and Shannon index metrics, and compared between groups using one-way analysis of variance (ANOVA) with Tukey's Honestly Significant Difference (HSD) post-hoc test. For analysis of beta diversity, principal coordinates analysis (PCoA) was performed at the OTU level using the weighted UniFrac measure and the statistical significance of the distance matrix was tested using permutational multivariate analysis of variance (PERMANOVA). Pairwise differences in beta diversity between GIT segments were calculated using beta-group-significance command implemented in QIIME2 v2021.11 [47]. For a variable to be considered having a significant influence on differences between groups in microbiota composition, a minimum R^2^ value of 0.25 and a *p* value less than 0.05 were both needed.

### Differentially abundant taxa between groups

To identify bacterial phyla and genera that differed significantly among GIT segments, and between the high and low FEC groups within GIT segment, the analysis of composition of microbiomes (ANCOM) procedure [48] was performed on the raw abundance data using the ANCOM v2.1 R script (https://github.com/FrederickHuangLin/ANCOM). Bacterial taxa present in less than 15% of samples were excluded from the analysis. We adjusted the GIT segment comparisons for FEC level. The *p* values were adjusted using the Benjamini–Hochberg procedure at the significance level of 0.05. Bacterial taxa with significant associations were declared by using ANCOM’s W-statistic with a threshold of 0.7 (Additional file 5: Table S5). For each bacteria that differed significantly between GIT segments, further pairwise comparison were performed based on centered log-ratio (CLR)-transformed abundance data using Wilcoxon signed rank test with *p* values adjusted using the Benjamini–Hochberg method. The results are available in Additional file 7: Table S7.

## Supplementary Information

**Additional file 1: Table S1**. Australian Sheep Breeding Values (ASBVs) for faecal egg count (FEC), worm counts and FEC of each animal used in the study.

**Additional file 2: Table S2**. V3-V4 amplicons of the 16S rRNA gene generated from each sample included in the experiment.

**Additional file 3: Table S3**. Results of Tukey’s test for post-hoc analysis of differences in microbial richness and Shannon index among gastro-intestinal tract (GIT) segments from High- and Low-FEC sheep.

**Additional file 4: Table S4**. OTU table and taxonomic information. For each sample, relevant metadata [i.e., gastrointestinal segment and experimental group (High- or Low-FEC)] can be obtained from Table S2.

**Additional file 5: Table S5**. ANCOM test used to the identification of bacterial phyla and genera showing significant differences among segments of the gastrointestinal tract, and between samples collected from High- and Low-FEC sheep.

**Additional file 6: Table S6.** Pairwise differences in beta diversity among GIT segments of High- and Low-FEC sheep.

**Additional file 7: Table S7.** Results of centered log-ratio (CLR) transformation using Wilcoxon signed rank test for each bacteria taxa that differed significantly among different GIT segments from High- and Low-FEC sheep.

## Data Availability

The raw sequencing reads generated in this study have been submitted to NCBI Sequence Read Archive (SRA) database under the BioProject accession number PRJNA674764.

## Abbreviations

ANCOM: Analysis of composition of microbiomes
ANOVA: Analysis of variance
ASBVs: Australian sheep breeding values
DPIRD: Department of primary industries and regional development
FEC: Faecal egg count
GIT: Gastro-intestinal tract
OTU: Operational taxonomic unit
PERMANOVA: Permutational multivariate analysis of variance
PCR: Polymerase chain reaction
PCoA: Principal coordinates analysis
SCFAs: Short-chain fatty acids
